# Association Between Pelvic Injury and Trauma-Induced Coagulopathy in Severe Trauma Patients: A Retrospective Single-Center Study

**DOI:** 10.3390/jcm15062365

**Published:** 2026-03-19

**Authors:** Tiphaine Pinasa, Pierre-Julien Cungi, Eric Meaudre, Michael Cardinale, Quentin Mathais

**Affiliations:** 1 Department of Anesthesiology and Intensive Care, Sainte Anne Military Teaching Hospital, 83000 Toulon, France; 2 French Armed Forces Academy (Académie de Santé des Armées), 75005 Paris, France

**Keywords:** pelvic injury, trauma-induced coagulopathy, severe trauma, hemorrhage, trauma management

## Abstract

**Background/Objectives**: Pelvic injuries are frequently associated with severe hemorrhage and may contribute to early trauma-induced coagulopathy (TIC). Whether pelvic injury is independently associated with TIC beyond overall injury severity remains unclear. The objective of this study was to evaluate the association between pelvic injury and TIC in severe trauma patients. **Methods**: We conducted a retrospective single-center study including adult severe trauma patients (injury severity score > 15) admitted between January 2012 and July 2020. Patients with moderate to severe traumatic brain injury (because of its specific coagulopathy and mortality), inter-hospital transfer, pregnancy, or long-term anticoagulant or antiplatelet therapy were excluded. Pelvic injury was defined as any traumatic lesion involving the pelvic girdle identified on admission computed tomography. TIC was defined by an international normalized ratio (INR) > 1.2 and/or fibrinogen < 1.5 g/L and/or platelet count < 100 G/L. Multivariable logistic regression was performed to identify factors associated with TIC. **Results**: Among 388 included patients (79.6% male, median age 39 years), 114 (29.4%) had a pelvic injury. TIC was present in 160 patients (41.3%), and TIC prevalence was significantly higher in patients with pelvic injury (*n* = 73–64.0%) compared to those without (*n* = 87–31.8%) (*p* < 0.001). After multivariate analysis, TIC was independently associated with pelvic injury (OR 2.81, 95% CI 1.63–4.89), shock index > 0.9 (OR 1.94, 95% CI 1.12–3.37), hemoglobin < 10 g/dL (OR 4.27, 95% CI 1.77–11.49), and lower base excess values on admission (OR per unit increase 0.92, 95% CI 0.87–0.97). Injury severity score and number of lesions (AIS ≥ 3) were not independently associated with TIC. **Conclusions**: Pelvic injury was independently associated with TIC after adjustment for injury severity, number of severe injuries, and markers of hemodynamic and metabolic shock, including shock index, hemoglobin level, and base excess. These findings suggest that patients with pelvic injury may represent a high-risk subgroup for early coagulopathy, supporting the need for early recognition and adapted resuscitation strategies. Further prospective studies are required to explore underlying mechanisms.

## 1. Introduction

Trauma remains a major global health burden, accounting for approximately 8% of worldwide deaths and over 36,000 annual fatalities in France [1,2]. Forty-two percent of trauma-related deaths occur in individuals under 50 years old, making trauma the leading cause of mortality in this age group [3]. Despite advances in trauma systems and resuscitation strategies, head injury and hemorrhage remain the main causes of trauma-related death, with uncontrolled bleeding being the leading cause of preventable mortality [4,5].

Trauma-induced coagulopathy (TIC) is a frequent and severe complication of major trauma, reported in approximately 25–40% of severely injured patients upon admission [6,7,8]. It represents a systemic failure of hemostatic mechanisms following trauma and manifests clinically as uncontrolled bleeding and biologically through abnormal coagulation parameters—prolonged prothrombin time (PT) and activated partial thromboplastin time, thrombocytopenia, and increased international normalized ratio (INR) [9,10]. TIC contributes to hemorrhagic shock, massive transfusion, multiple organ failure, collateral infections and longer intensive care unit (ICU) stays [11].

Pelvic injuries represent a distinct subset of traumatic lesions, most often resulting from high-energy mechanisms and frequently associated with severe hemorrhage [12,13]. Although pelvic injuries account for a relatively small proportion of all skeletal fractures, they carry substantial morbidity and mortality, particularly in the presence of hemodynamic instability [12,13,14,15,16]. The anatomical characteristics of the pelvis—including extensive cancellous bone, large venous plexuses, and major vascular structures—allow for significant blood loss into the retroperitoneal space [17]. Accordingly, coagulopathy has been reported in approximately 25–50% of patients with pelvic trauma in previous studies [18,19]. However, these estimates vary widely depending on the definitions of coagulopathy used, the timing of biological assessment, and the characteristics of the studied populations, which range from isolated pelvic fractures to heterogeneous polytrauma cohorts. As a result, direct comparison of coagulopathy prevalence across studies remains challenging.

While the association between severe trauma and trauma-induced coagulopathy is well established, the specific contribution of pelvic injury to early coagulopathy remains unclear. Pelvic trauma is closely intertwined with several recognized drivers of TIC, including hemorrhage, shock, tissue damage, and inflammatory activation; however, whether pelvic injury is independently associated with TIC beyond overall injury severity and hemodynamic derangement has not been clearly established in severe trauma patients, to our knowledge.

The primary objective of this study was to assess the association between pelvic injury and TIC in a cohort of severe trauma patients. The secondary objective was to identify clinical and biological factors independently associated with the occurrence of TIC in the overall study population.

## 2. Materials and Methods

### 2.1. Design

This was an observational, retrospective, single-center cohort study conducted at Sainte-Anne Military Teaching Hospital (Toulon, France), a Level 1 trauma center. All consecutive adult severe trauma patients admitted between January 2012 and July 2020 were screened for inclusion.

During the study period, institutional trauma management protocols, including damage control resuscitation principles and early hemorrhage control strategies, remained globally consistent. Data were analyzed retrospectively without any intervention in patient management.

### 2.2. Data Sources and Ethical Approval

All trauma patients admitted to the emergency department were prospectively recorded in a local trauma database, in accordance with French data protection regulations. The database was registered with the Commission Nationale de l’Informatique et des Libertés (CNIL), the French national data protection authority (registration number: 2002878v0). The study protocol was approved by the local ethics committee (approval reference: 11873-2021-04). Due to the retrospective and observational nature of the study, informed consent was waived.

Collected data included demographics, mechanism of injury, prehospital physiological parameters and resuscitation measures, initial emergency department management, radiological findings, interventional procedures (surgery and embolization), laboratory results on admission, transfusion requirements within the first 24 h, ICU stay, and in-hospital outcomes.

According to our institutional trauma management protocol, patients were categorized into three groups during initial hospital management in the trauma bay. Group 1 included hemodynamically unstable patients, defined by severe hypotension (systolic blood pressure (SBP) < 65 mmHg) associated with ongoing active bleeding. Group 2 comprised patients who were initially unstable but achieved hemodynamic stabilization after the initial phase of resuscitation in the trauma bay, with SBP > 90 mmHg. Group 3 consisted of hemodynamically stable patients, defined by SBP > 90 mmHg without the need for vasopressor support.

The following scores were calculated: ISS (injury severity score), MGAP (Mechanism, Glasgow coma scale, Age and Arterial Pressure), TASH (trauma-associated severe hemorrhage), TRISS (trauma injury severity score), RTS (revised trauma score), SAPS II (Simplified Acute Physiology Score) and SI (shock index).

### 2.3. Inclusion and Exclusion Criteria

All adult trauma patients meeting the criteria for severe trauma were eligible for inclusion. Severe trauma was defined as an injury severity score (ISS) >15 and at least one positive Vittel triage criterion [20]. The Vittel criteria are French prehospital triage guidelines including physiological parameters (systolic blood pressure < 90 mmHg, SpO_2_ < 90%, Glasgow Coma Scale score < 13), anatomical lesions (penetrating trauma to specific regions, severe fractures, major hemorrhage, etc.), injury mechanisms (ejection from a vehicle, fall from a height > 6 m, blast injury, etc.) and prehospital resuscitation requirements (assisted ventilation, administration of vasopressors, etc.).

Exclusion criteria included age <18 years, inter-hospital transfer, pregnancy, moderate to severe traumatic brain injury (AIS for the head ≥3), pre-injury anticoagulant or antiplatelet therapy, and missing key variables required for the analysis, particularly pelvic injury status and admission coagulation parameters. Information regarding inclusion and exclusion criteria was collected from the severe trauma registry and completed using the patient medical record throughout the hospital stay.

Patients with moderate to severe traumatic brain injury were excluded because of the specific coagulopathy associated with brain injury, which could confound the assessment of TIC related to extracranial injuries and contribute to high mortality [21,22].

### 2.4. TIC and Pelvic Injury Definitions

Trauma-induced coagulopathy (TIC) was defined by the presence of at least one of the following biological abnormalities measured on admission to the emergency department: international normalized ratio (INR) > 1.2, fibrinogen level < 1.5 g/L, or platelet count < 100 G/L. This admission-based definition was chosen to reflect early clinically relevant coagulation abnormalities. Although no universal biological definition of TIC exists, an INR > 1.2 is commonly used to define trauma-related coagulopathy, as supported by the European guidelines on the management of major bleeding and coagulopathy following trauma [23]. The thresholds for fibrinogen and platelet count were also selected pragmatically, in line with these guidelines and with prior trauma literature, to capture clinically meaningful early coagulation disorders [23,24,25]. Biological samples were obtained at emergency department admission, as part of the initial trauma evaluation, prior to any surgical or radiological hemostatic procedures. According to institutional protocol, blood sampling is typically performed within the first 10 min following patient arrival in the emergency department.

Pelvic injury was defined as any traumatic lesion involving the pelvic girdle identified on the admission computed tomography scan. This definition included pelvic ring injuries as well as sacral or iliac fractures (including isolated acetabular fractures), and symphyseal or sacroiliac disruptions. Pelvic injuries were secondarily classified according to the Tile classification to describe pelvic ring stability, including stable (Tile A), rotationally unstable (Tile B), and vertically unstable (Tile C) patterns. Classification was performed by a trauma intensivist based on a review of radiology reports interpreting the initial CT imaging and was conducted blind to clinical outcomes and laboratory data [26,27]. No formal inter-rater reliability assessment was performed, and potential misclassification related to retrospective classification is acknowledged.

### 2.5. Statistical Analysis

Analyses were performed using R software version 4.0.5. Analysis of missing values was done using the R naniar package version 0.6.1. Quantitative variables were described as mean ± SD or median [IQR], as appropriate, and compared using the *t*-test or Wilcoxon–Mann–Whitney test. Qualitative variables were compared using the χ^2^ test with Yates’s correction or Fisher’s exact test. Clinically relevant variables with *p* < 0.2 in univariate analysis were entered into a multivariate logistic regression. To evaluate the impact of collinearity, we computed the Variable Inflation Factor. To improve clinical interpretability of the multivariable model, continuous variables were discretized prior to analysis. When established clinical thresholds were available in the literature, these predefined cut-points were used. Otherwise, optimal cut-points were identified using a data-driven approach based on a genetic algorithm (CatPredi R package, version 1.1) [28]. This approach was chosen to enhance model readability and to facilitate clinical understanding of predictor effects, while maintaining the predictive performance of the logistic regression model. These cut-points were intended for analytical and interpretative purposes and should not be considered definitive clinical thresholds for patient management. After the transformation of variables, we realized a logistic regression to obtain the final model. Model validity was assessed by the Hosmer–Lemeshow test and area under the ROC curve. A *p*-value <0.05 was considered statistically significant. Graphical representation of the multivariate analysis was realized with the forestmodel package 0.6.2.

## 3. Results

### 3.1. Patient Characteristics

**Figure 1:**
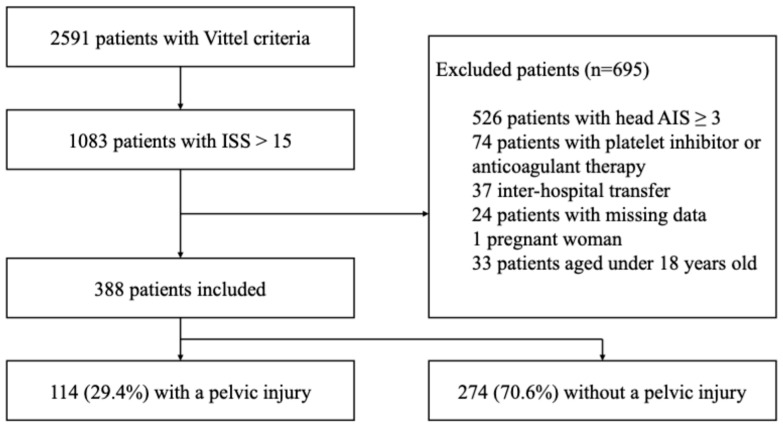
Flow chart of the study.

During the study period, 2591 trauma patients meeting Vittel criteria were admitted to the emergency department. Among them, 1083 patients had severe trauma (ISS > 15). A total of 695 patients were excluded for the following reasons: moderate to severe traumatic brain injury (AIS head ≥ 3, *n* = 526), pre-injury anticoagulant or antiplatelet therapy (*n* = 74), secondary transfer (*n* = 37), insufficient data quality (*n* = 24), pregnancy (*n* = 1), and age < 18 years (*n* = 33). Finally, 388 patients were included in the analysis, of whom 114 (29.4%) had a pelvic injury and 274 (70.6%) did not (Figure 1). Of the 388 patients and 116 variables assessed, the overall missingness was 5.82%, with over two-thirds of the variables affected. Notably, severe missingness was confined to a small subset, predominantly mortality indicators and laboratory or injury-specific variables, where absent data reflected logical clinical scenarios—such as death-related fields only completed for deceased patients and certain tests or classifications applied selectively (Figure S1 and Table S1).

In our study, 309 patients (79.6%) were male, with a median age of 39 years [27,28,29,30,31,32,33,34,35,36,37,38,39,40,41,42,43,44,45,46,47,48,49,50,51,52,53]. Motorcycle crashes were the leading cause of trauma (47.2%). Prehospital data are available in Table 1.

Twenty-one (5.5%) patients presented with SBP < 65 mmHg, and 70 (18%) had SBP between 65 and 90 mmHg on arrival. The median ISS was 24 [18,19,20,21,22,23,24,25,26,27,28,29]. Eight (2.6%) underwent resuscitative thoracotomy; 135 (34.8%) required emergency hemostatic surgery; and 44 (11.3%) underwent embolization. One hundred eighty-two (46.9%) were admitted to the ICU, and mortality was 6.2%. Hospital data are available in Table 2. Overall, 196 patients (50.5%) received at least one blood product within the first 24 h.

### 3.2. Characteristics of the Population with Pelvic Injury

One hundred fourteen (29.4%) patients had pelvic injury: 77 (67.5%) had type A, 17 (14.9%) type B, and 20 (17.5%) type C. A pelvic binder was applied in 89 (22.9%) patients overall, including 81% of those with Tile B or C lesions. Patients with pelvic injury had higher ISS and SI, more frequent embolization (19.3% vs. 8%, *p* < 0.001), and greater transfusion requirements. The median red blood cell transfusion during the first 24 h was 2 [0–5] units in the pelvic group versus 0 [0–2] in the non-pelvic group (*p* < 0.001) (Table 2). ICU admission and length of stay were significantly longer, and mortality was higher (11.4% vs. 4.0%, *p* = 0.012) (Table 2).

Among patients with pelvic injury, 28 (24.6%) underwent specific hemostatic surgery and/or embolization focused on the pelvis (e.g., pelvic external fixation, retroperitoneal packing, hypogastric artery embolization). Fourteen (18.2%) patients with Tile A pelvic injury, 6 (35.3%) with Tile B and 8 (40%) with Tile C needed an emergent surgery and/or embolization.

Patients with pelvic injury more frequently required blood product transfusion within the first 24 h compared with patients without pelvic injury (73.7% vs. 40.9%, *p* < 0.001). Among the transfused patients, those with pelvic injury received significantly higher volumes of red blood cells and total blood products than patients without pelvic injury (Table 2).

### 3.3. Characteristics of the Population with TIC

TIC was present in 160 (41.3%) patients in the overall cohort. Among patients meeting the definition of TIC, INR elevation (INR > 1.2) was present in all cases. Most patients met a single TIC criterion based on INR alone (*n* = 118), whereas a smaller proportion met multiple criteria, including INR elevation associated with hypofibrinogenemia (*n* = 35), thrombocytopenia (*n* = 2), or both hypofibrinogenemia and thrombocytopenia (*n* = 5). The prevalence of TIC was significantly higher in patients with pelvic injury, affecting 73 of 114 patients (64.0%), compared with 87 of 274 patients (31.8%) without pelvic injury (*p* < 0.001). The distribution between categories A, B and C of the Tile classification found TIC in 11 (68.8%) patients categorized as Tile B, 49 (63.6%) patients categorized as type A and 13 (65%) patients categorized as type C compared to 87 (31.8%) patients free of pelvic trauma (*p* < 0.001). No significant difference in TIC rate was found between Tile A, B and C lesions (*p* = 0.74).

Among transfused patients, TIC was associated with markedly higher transfusion requirements. Patients with TIC received significantly greater volumes of red blood cells, plasma, and total blood products compared with transfused patients without TIC (Table 3). Transfusion requirements increased further when pelvic injury and TIC were combined. Among transfused patients, those presenting both pelvic injury and TIC had the highest blood product consumption, particularly in terms of red blood cells and total blood products (Table 3).

Among patients with pelvic injury, trauma-induced coagulopathy was frequent across all Tile classifications, with no significant difference in TIC prevalence between Tile A, B, and C injury patterns. However, increasing pelvic injury severity according to Tile classification was associated with more severe metabolic acidosis and a greater need for hemostatic interventions. These results are detailed in Table S2.

Table 4 and Table 5 present the results of the univariate analysis.

### 3.4. Multivariate Analysis

**Figure 2:**
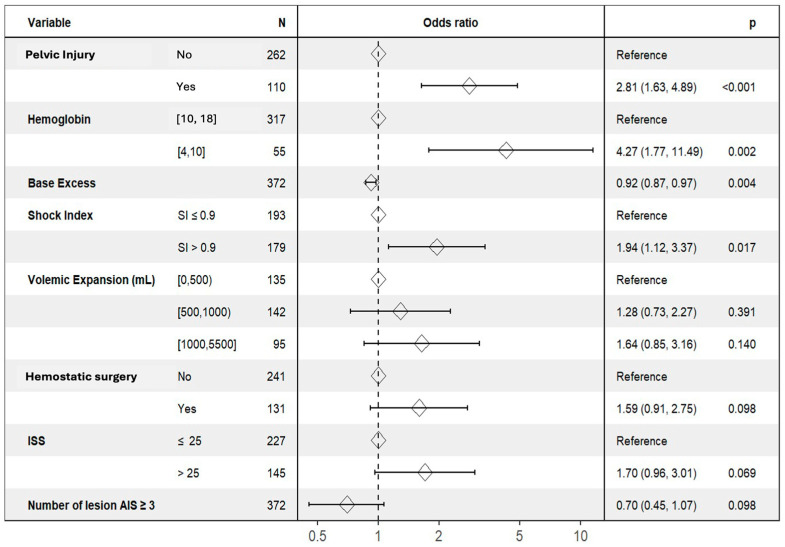
Forest plot of independent factors associated with trauma-induced coagulopathy in severe trauma patients (ISS > 15).

A multivariate analysis was performed to identify factors associated with TIC. This analysis was conducted on 372 complete cases. Exclusions due to missing data (*n* = 16) were primarily related to missing admission laboratory or clinical variables, including base excess on admission (*n* = 10), shock index due to missing systolic blood pressure or heart rate at admission (*n* = 3), hemoglobin measurement on admission (*n* = 1), ISS (*n* = 1), and missing TIC status on admission (*n* = 1). The choice of a >0.9 threshold for shock index was based on previous studies [29,30,31]. Independent predictors of TIC were pelvic injury (OR 2.81, 95% CI 1.63–4.89), shock index > 0.9 (OR 1.94, 95% CI 1.12–3.37), hemoglobin level < 10 g/dL (OR 4.27, 95% CI 1.77–11.49), and lower base excess values on admission (OR per unit increase 0.92, 95% CI 0.87–0.97). ISS, volemic expansion, the need for hemostatic surgery, and number of AIS ≥ 3 lesions were not independently associated with TIC (Table 6 and Figure 2).

## 4. Discussion

### 4.1. Summary of Main Findings

In this retrospective single-center cohort of severe trauma patients, pelvic injury was associated with a high prevalence of trauma-induced coagulopathy on admission. After multivariable adjustment, pelvic injury remained associated with TIC, with an approximately 2.8-fold increase in odds compared with patients without pelvic injury. This association persisted after multivariable adjustment for injury severity and early physiological markers of shock, including shock index, hemoglobin level, and base excess, all of which were also independently associated with TIC.

Importantly, the prevalence of TIC did not differ significantly across pelvic injury patterns according to the Tile classification, suggesting that mechanical instability alone does not fully explain the observed association.

### 4.2. Comparison with Existing Literature

In major epidemiological studies reporting pelvic injury patterns according to the Tile classification, the distribution of Tile A, B, and C injuries varies considerably across cohorts [32,33,34,35]. Such variability likely reflects differences in study populations and inclusion criteria. In particular, the exclusion of moderate to severe traumatic brain injury in the present cohort may partly explain differences in pelvic injury severity profiles, as high-energy mechanisms are frequently associated with concomitant brain injury. In this study, although more severe pelvic injury patterns were associated with more severe metabolic acidosis and a greater need for hemostatic surgeries, TIC prevalence did not differ significantly across Tile classifications. This finding suggests that mechanical instability alone might not fully account for the occurrence of early coagulopathy in patients with pelvic injury.

Previous studies have reported TIC prevalence ranging from 25% to 50% in pelvic trauma, with substantial heterogeneity in patient populations, definitions of coagulopathy, and timing of biological assessment [18,19]. The higher prevalence observed in the present study likely reflects the focus on severely injured patients and the use of a pragmatic, admission-based definition of TIC. These differences complicate direct comparisons but emphasize the relevance of early coagulation abnormalities in this high-risk population.

Pelvic injury is closely associated with several recognized drivers of TIC, including hemorrhage, hypoperfusion, and tissue injury. Although pelvic trauma may therefore represent a marker of global injury severity, the persistence of the association after multivariable adjustment suggests that pelvic injury identifies a subgroup of trauma patients at particularly high risk of early coagulopathy.

### 4.3. Potential Mechanisms Linking Pelvic Trauma and TIC

Hemostasis is a complex process that involves three phases: primary (platelet aggregation), secondary (clot formation), and fibrinolysis (clot dissolution) [36]. TIC arises from multiple interacting mechanisms—protein C activation, endothelial dysfunction, inflammation, platelet over-activation, and fibrinogen depletion [9,22,24,37,38,39]. Contributory factors include dilution, hypothermia, acidosis, endotheliopathy and pre-existing bleeding disorders [40]. Several mechanisms could plausibly contribute to the association between pelvic injury and TIC.

#### 4.3.1. Hemorrhage and Hemostatic Factor Consumption: Necessary but Not Sufficient?

Massive hemorrhage is the most intuitive mechanism linking pelvic trauma to early coagulopathy. Pelvic fractures may generate substantial bleeding from cancellous bone, venous plexuses (≈85%), or arterial lesions (≈15%), with exsanguination severity influenced by vessel involvement and sacroiliac ligament disruption opening the retroperitoneal space [16,19]. However, in our cohort, massive pelvic bleeding alone cannot account for the high prevalence of TIC. Only 22 patients (19.3%) required arterial embolization, and 48 (42.1%) underwent emergency surgery, while just 28 patients (24.6%) needed a pelvic-focused hemostatic intervention. Early pelvic binder use in unstable fractures (81%) likely helped reduce blood loss and transfusion needs [41]. Despite this, pelvic trauma remained strongly associated with TIC, including in patients without major hemorrhage requiring operative control.

In addition to overt bleeding, coagulation factor consumption and dilutional effects contribute to early TIC. Loss of ≥25% of circulating coagulation factors during hemorrhage, combined with even moderate crystalloid administration, may precipitate clotting abnormalities [42]. Although modern resuscitation strategies favor restricted fluids and permissive hypotension, prehospital volumes in our cohort (median 500 mL) could still exacerbate dilutional coagulopathy. Moreover, many patients with pelvic injury presented with systolic blood pressure <100 mmHg and required early transfusion, reflecting a degree of hypovolemia and hypoperfusion sufficient to promote coagulation factor depletion.

Overall, while hemorrhage and dilution contribute to TIC, neither the severity of bleeding nor the intensity of fluid resuscitation fully explains the high rate observed in pelvic trauma, suggesting the involvement of additional mechanisms.

#### 4.3.2. Tissue Factor-Driven Activation of Coagulation: A Plausible Biological Driver

A second mechanism involves tissue factor (TF) exposure at the site of injury. TF and activated factor VII interactions trigger extrinsic coagulation pathway overactivation, leading to rapid consumption of clotting factors. Mesenchymal stem cells from bone marrow and adipose tissue express significant amounts of TF and exhibit procoagulant activity when in contact with whole blood [43,44]. Experimental models even show disseminated intravascular coagulation after infusion of bone marrow-derived stem cells [45]. Since pelvic bones contain approximately one-third of the body’s hematopoietic marrow volume, pelvic trauma may release large quantities of TF-rich material into the circulation [46]. This hypothesis is consistent with the markedly elevated fibrin monomer levels (>150 µg/mL) found in our pelvic injury group, indicating a possible massive extrinsic pathway activation [47].

#### 4.3.3. Endotheliopathy of Trauma: A Systemic Amplifier?

Finally, systemic endotheliopathy may contribute to pelvic injury-associated TIC. The vascular endothelium plays a key role in maintaining blood–vascular homeostasis. Severe trauma triggers endothelial activation, glycocalyx shedding, inflammatory cytokine release, increased endothelial permeability, and dysregulated hemostasis [24,48]. Hypoperfusion, hypoxia, tissue damage, and catecholamine surge amplify these mechanisms, promoting platelet dysfunction, hyperfibrinolysis, and alternating pro- and anticoagulant responses. Syndecan-1, a circulating marker of glycocalyx degradation, is consistently elevated in patients with endotheliopathy-driven TIC and could represent a useful biomarker to further characterize this mechanism in pelvic trauma [49,50,51].

However, these mechanisms were not directly assessed in the present study. The absence of mechanistic biomarkers, such as tissue factor activity, markers of endothelial injury, or viscoelastic parameters, limits the ability to formally test these hypotheses and to delineate the relative contribution of each pathway. Accordingly, the present findings should be interpreted as supporting the hypothesis of a pelvic-associated TIC phenotype rather than demonstrating a distinct or specific coagulopathy mechanism.

### 4.4. Clinical Implications

These observations carry several practical consequences.

#### 4.4.1. Early Identification of TIC in Pelvic Trauma

Given the high prevalence of TIC—even in patients without massive hemorrhage—clinicians might consider maintaining a low threshold for early coagulation testing and point-of-care viscoelastic monitoring. As with traumatic brain injury, pelvic trauma may identify a subgroup of patients at increased risk of early coagulopathy, without implying a shared underlying mechanism.

#### 4.4.2. Reinforcing Damage Control Resuscitation (DCR) Principles

Optimal management should include restrictive crystalloid use, permissive hypotension (in the absence of TBI), early balanced transfusion, prevention of hypothermia, acidosis, and hypocalcemia, and rapid control of bleeding sources with shortened hemostatic surgical or angiographic procedures [52,53,54]. Given the unique risk profile of pelvic trauma, hemostatic resuscitation may be warranted even before overt shock develops.

#### 4.4.3. Orthopedic Damage Control (ODC) for Pelvic Injuries

ODC is meant to stabilize a potentially hemorrhagic fracture but to avoid a prolonged early reconstructive surgery that would constitute a second aggression, with systemic inflammatory complications [55]. Pelvic stabilization through packing, external fixation, transcondylar traction in the case of vertical dislocation, or balloon/tamponade strategies remains central to early care [56]. In the absence of a hybrid trauma room, patients without hemodynamic impairment or those stabilized using damage control resuscitation may benefit from prior embolization before considering surgical stabilization. Unstable patients, despite initial resuscitation, may immediately undergo ODC as described above. Definitive surgery must be performed when the pelvis is unstable, either for open-book or Tile C lesions. Ideally, it should be performed between days 4 and 10 to minimize the “second hit” phenomenon [55,57,58].

#### 4.4.4. System-Level Implications

Finally, these data suggest that in severe trauma patients, there is no such thing as a “minor” pelvic injury. These lesions often reflect high-energy mechanisms and were associated in this study with a markedly increased prevalence of trauma-induced coagulopathy, beyond what could be explained by hemorrhage severity or mechanical instability alone [59]. The management of these patients, including ODC and DCR, can be complex and require specific skills. The management of severe pelvic trauma is complex and frequently requires a coordinated multidisciplinary approach combining damage control resuscitation, orthopedic damage control, interventional radiology, and definitive surgical management. Several studies and guidelines emphasize that optimal outcomes in patients with severe pelvic injuries rely on early access to specialized trauma care, including pelvic stabilization techniques, angiographic embolization, and massive transfusion capabilities, which are typically available in high-level trauma centers [60,61,62].

### 4.5. Limitations

This study has several limitations that should be acknowledged. First, its retrospective, single-center design inherently limits causal inference and exposes the results to potential selection and information biases. The restriction to patients with severe trauma (ISS > 15), as well as the exclusion of patients with moderate to severe traumatic brain injury and those receiving pre-injury anticoagulant or antiplatelet therapy, may limit the generalizability of our findings, particularly to older trauma populations and to real-world polytrauma settings. The exact time interval between injury and blood sampling, as well as the precise impact of early prehospital interventions (fluids, blood products, tranexamic acid), could not be reliably captured and may have influenced early coagulation parameters. Finally, although institutional trauma resuscitation protocols remained globally consistent over the study period, minor temporal evolutions—such as increasing use of tranexamic acid—may have occurred and could have had a modest influence on early coagulation profiles.

Second, patients with pelvic injury in our cohort were more severely injured overall and presented with greater hemodynamic impairment on emergency department admission, which raises the possibility of confounding by injury severity. Although multivariable adjustment was performed to account for established markers of injury severity and shock, residual confounding cannot be excluded, as not all clinically relevant factors can be fully captured in an observational study. In addition, adjustment for variables that may lie on the causal pathway between pelvic injury and TIC could have biased effect estimates toward the null. The multivariable model included both a priori confounders (ISS and number of severe injuries) and variables potentially on the causal pathway between pelvic injury and TIC (shock index, hemoglobin level, fluid volume, and damage control surgery). Finally, categorization of continuous variables such as hemoglobin level, ISS or SI may have resulted in information loss and could limit generalizability to other populations. Therefore, the present results should be interpreted as describing associations rather than demonstrating causality or a distinct pelvic-specific coagulopathy mechanism.

Third, biological samples were obtained at emergency department admission, but the exact time interval between injury and sampling was not reliably recorded, and some samples may have been drawn during the very early resuscitation phase due to the simultaneous nature of trauma care. This may have introduced variability in the assessment of early coagulation abnormalities.

Finally, key mechanistic biomarkers relevant to trauma-induced coagulopathy—including tissue factor activity, thrombin generation, protein C pathway markers, endothelial injury biomarkers, and viscoelastic parameters—were not available. The absence of these data limits the ability to explore underlying biological mechanisms and to determine whether pelvic injury is associated with a distinct pathophysiological phenotype or reflects the combined effects of severe tissue injury and hemorrhagic shock.

Future prospective studies should incorporate standardized timing of biological sampling, dynamic coagulation monitoring (including viscoelastic testing), and mechanistic biomarkers to better characterize the relationship between pelvic injury and trauma-induced coagulopathy and to improve the accuracy and applicability of these findings.

## 5. Conclusions

In this retrospective single-center cohort study of severe trauma patients, pelvic injury was associated with a high prevalence of TIC on admission to the emergency department. TIC occurred in 64.0% of patients with pelvic injury and was approximately 2.8-fold more frequent than in patients without pelvic injury after multivariable adjustment.

Pelvic injury remained independently associated with TIC after adjustment for injury severity, number of severe injuries, and early physiological markers of shock, including shock index, hemoglobin level, and base excess. These findings indicate that pelvic injury identifies a subgroup of severe trauma patients at particularly high risk of early coagulopathy, rather than demonstrating a causal relationship.

Although the prevalence of TIC appeared similar across pelvic injury patterns and could suggest that mechanical instability alone does not fully explain this association, the absence of formal comparative analyses and direct mechanistic biomarker measurements precludes definitive conclusions regarding the role of fracture stability or underlying pathophysiological mechanisms. Further prospective multicenter studies incorporating dynamic coagulation assessment and mechanistic biomarkers are needed to better clarify the respective contributions of hemorrhage, tissue injury, and endothelial dysfunction to TIC in patients with pelvic injury.

## Figures and Tables

**Table 1:** Population characteristics and prehospital data. SBP: systolic blood pressure; SpO_2_: oxygen saturation; GCS: Glasgow coma scale, TXA: tranexamic acid.

	All Patients *n* = 388	Patients Without Pelvic Injury *n* = 274	Patients with Pelvic Injury *n* = 114	*p*
Age (med, [IQR])	39.0 [27.0;53.0]	38.0 [26.0;51.8]	41.0 [29.0;55.0]	0.185
Gender (*n*, %)				
Male	309 (79.6%)	231 (84.3%)	78 (68.4%)	0.001
Trauma mechanism (*n*, %)				
Fall < 3 m	3 (0.77%)	3 (1.09%)	0 (0.00%)	0.559
Fall between 3 and 6 m	15 (3.87%)	10 (3.65%)	5 (4.39%)	0.775
Fall > 6 m	33 (8.51%)	12 (4.38%)	21 (18.4%)	<0.001
Car road accident	75 (19.3%)	57 (20.8%)	18 (15.8%)	0.318
Pedestrian road accident	19 (4.90%)	8 (2.92%)	11 (9.65%)	0.011
Bike road accident	11 (2.84%)	9 (3.28%)	2 (1.75%)	0.520
Motorcycle road accident	183 (47.2%)	133 (48.5%)	50 (43.9%)	0.466
Stab	17 (4.38%)	17 (6.20%)	0 (0.00%)	0.004
Gunshot	15 (3.87%)	14 (5.11%)	1 (0.88%)	0.078
Physiological data				
Heart rate (med, [IQR], bpm)	92.0 [80.0;116]	90.0 [80.0;115]	100 [85.2;120]	0.016
SBP (med, [IQR], mmHg)	110 [90.0;128]	112 [95.8;130]	104 [86.5;120]	0.001
SpO_2_ (med, [IQR], %)	98.0 [95.0;100]	98.0 [94.5;100]	99.0 [95.0;100]	0.625
Respiratory rate (med, [IQR], cycles/min)	20.0 [16.0;22.8]	20.0 [16.0;24.0]	16.0 [15.0;20.0]	0.102
GCS (med, [IQR])	15.0 [14.0;15.0]	15.0 [15.0;15.0]	15.0 [14.0;15.0]	0.124
Prehospital protocol				
Crystalloid volume (med, [IQR], mL)	500 [250;750]	500 [250;750]	500 [250;900]	0.354
Intubation (*n*, %)	70 (18.0%)	44 (16.1%)	26 (22.8%)	0.153
TXA (*n*, %)	115 (29.6%)	80 (29.2%)	35 (30.7%)	0.862
Pelvic binder (*n*, %)	89 (22.9%)	18 (6.57%)	71 (62.3%)	0.001

**Table 2:** Hospital data. SBP: systolic blood pressure; SpO_2_: oxygen saturation; GCS: Glasgow coma scale; TXA: tranexamic acid; FAST: focused abdominal sonography for trauma; RBC: red blood cell; ICU: intensive care unit.

	All Patients *n* = 388	Patients Without Pelvic Injury *n* = 274	Patients with Pelvic Injury *n* = 114	Significance Level (*p*)
Physiological parameters in emergency room				
Heart rate (med, [IQR], bpm)	95.0 [82.0;117]	92.0 [81.0;110]	106 [85.0;124]	0.001
SBP (med, [IQR], mmHg)	110 [90.0;125]	111 [95.0;127]	98.5 [81.2;117]	<0.001
SpO_2_ (med, [IQR], %)	100 [97.0;100]	100 [97.0;100]	100 [97.0;100]	0.430
Respiratory rate (med, [IQR], cycles/min)	20.0 [17.0;24.8]	20.0 [17.0;24.0]	20.0 [18.0;25.0]	0.843
GCS (med, [IQR])	15.0 [15.0;15.0]	15.0 [15.0;15.0]	15.0 [14.0;15.0]	0.001
Cardiac arrest (*n*, %)	10 (2.58%)	7 (2.55%)	3 (2.63%)	1.000
Emergency room protocol (n, %)				
TXA	99 (25.5%)	56 (20.4%)	43 (37.7%)	0.001
Vasopressors	110 (28.4%)	66 (24.1%)	44 (38.6%)	0.006
IOT	62 (16.0%)	41 (15.0%)	21 (18.4%)	0.487
Thoracotomy	8 (2.06%)	7 (2.55%)	1 (0.88%)	0.446
Positive abdominal FAST (*n*, %)	71 (18.3%)	50 (18.2%)	21 (18.4%)	1.000
Hemodynamic status (SBP in ER) (*n*, %)				
>90 mmHg	295 (76.4%)	219 (80.5%)	76 (66.7%)	<0.001
[65–90] mmHg	70 (18.1%)	42 (15.4%)	28 (24.6%)	<0.001
≤65 mmHg	21 (5.44%)	11 (4.04%)	10 (8.77%)	<0.001
Scores				
MGAP (med, [IQR])	23.0 [18.0;25.0]	23.0 [20.0;25.0]	22.0 [15.0;23.0]	<0.001
RTS (med, [IQR])	7.84 [7.55;7.84]	7.84 [7.84;7.84]	7.84 [7.11;7.84]	0.001
TRISS (med, [IQR])	3.63 [2.55;4.22]	3.80 [2.73;4.22]	3.05 [2.01;4.05]	0.001
TASH (med, [IQR])	5.00 [1.00;11.0]	4.00 [1.00;7.75]	11.0 [7.00;17.0]	<0.001
IGS 2 (med, [IQR])	20.0 [13.0;32.0]	19.0 [11.0;29.0]	24.0 [15.0;41.0]	<0.001
ISS (med, [IQR])	24.0 [18.0;29.0]	22.0 [18.0;29.0]	25.0 [21.0;34.0]	0.002
SI > 0.9 (*n*, %)	182 (47.3%)	110 (40.6%)	72 (63.2%)	<0.001
Interventional management (*n*, %)				
Hemostatic surgery	135 (34.8%)	87 (31.8%)	48 (42.1%)	0.067
Surgery in 24 h	222 (57.2%)	151 (55.1%)	71 (62.3%)	0.235
Embolization	44 (11.3%)	22 (8.03%)	22 (19.3%)	0.003
Transfusion requirements during the first 24 h				
Any blood product transfusion (*n*, %)	196 (50.5)	112 (40.9)	84 (73.7)	<0.001
Units transfused (med, [IQR]) *	7 [3–14]	5 [2–13]	8 [4–16]	0.034
≥1 RBC transfusion (*n*, %)	192 (49.5)	110 (40.1)	82 (71.9)	<0.001
RBC units transfused (med, [IQR]) *	5 [2–10]	4 [2–8.25]	7 [4–10.2]	0.041
≥1 plasma transfusion (*n*, %)	138 (35.8)	75 (27.7)	63 (55.3)	<0.001
Plasma units transfused (med, [IQR]) *	2 [0–4]	1 [0–4]	2 [0–4]	0.131
≥1 platelet transfusion (*n*, %)	35 (9.1)	18 (6.6)	17 (14.9)	0.017
Platelet units transfused (med, [IQR]) *	0 [0–0]	0 [0–0]	0 [0–0]	0.513
Evolution				
ICU admission (*n*, %)	182 (46.9%)	114 (41.6%)	68 (59.6%)	0.002
ICU length of stay (med, [IQR], days)	0.00 [0.00;4.00]	0.00 [0.00;3.00]	1.00 [0.00;6.00]	0.002
Death (*n*, %)	24 (6.19%)	11 (4.01%)	13 (11.4%)	0.012

* Median [IQR] calculated among patients receiving at least one unit of the corresponding blood product.

**Table 3:** Blood product consumption according to pelvic injury and trauma-induced coagulopathy. TIC: trauma-induced coagulopathy; RBC: red blood cell.

Group	*n*	RBC Units, Med, [IQR]	Plasma Units, Med, [IQR]	Platelet Units, Med, [IQR]	Total Blood Products, Med, [IQR]
No pelvic injury + No TIC	187	0.00 [0.00;0.75]	0.00 [0.00;0.00]	0.00 [0.00;0.00]	0.00 [0.00;1.00]
Pelvic injury + No TIC	41	1.00 [0.00;5.25]	0.00 [0.00;2.00]	0.00 [0.00;0.00]	1.00 [0.00;5.75]
No pelvic injury + TIC	87	4.00 [0.00;9.75]	4.00 [0.00;8.00]	0.00 [0.00;0.00]	4.00 [0.00;14.0]
Pelvic injury + TIC	73	5.00 [3.00;10.0]	4.00 [0.00;8.00]	0.00 [0.00;0.00]	7.00 [4.00;16.0]
*p* value (overall)		<0.001	<0.001	-	<0.001

**Table 4:** Coagulation parameters comparison (patients with and without pelvic lesion). PT: prothrombin time; TQ: quick time; INR: international normalized ratio; aPTT: activated partial thomboplastin time.

	All Patients *n* = 388	Patients Without Pelvic Injury *n* = 274	Patients with Pelvic Injury *n* = 114	Significance Level (*p*)
Biological results at admission				
Coagulopathy (*n*, %)	160 (41.3%)	87 (31.8%)	73 (64.0%)	<0.001
PT (med, [IQR], %)	79.0 [67.5;89.0]	83.0 [72.0;91.0]	71.0 [57.0;80.8]	<0.001
TQ Quick time (med, [IQR], s)	14.9 [14.0;16.4]	14.6 [13.8;15.7]	16.0 [14.8;18.0]	<0.001
INR (med, [IQR])	1.13 [1.08;1.29]	1.10 [1.06;1.20]	1.22 [1.13;1.40]	<0.001
aPTT (med, [IQR], s)	31.0 [29.0;35.0]	31.0 [29.0;33.0]	34.0 [30.0;39.0]	<0.001
raPTT (med, [IQR])	0.97 [0.90;1.10]	0.91 [0.90;1.00]	1.00 [0.90;1.20]	<0.001
Fibrinogen (med, [IQR], g/L)	2.30 [1.90;2.78]	2.38 [1.98;2.87]	2.15 [1.65;2.57]	0.001
Platelet count (med, [IQR], G/L)	226 [187;273]	234 [192;277]	216 [184;259]	0.020
Fibrin monomers (med, [IQR], µg/mL)	135 [48.8;151]	97.5 [34.8;151]	151 [136;151]	<0.001
D-dimers (med, [IQR], µg/dL)	9.00 [4.76;18.2]	6.24 [3.44;17.7]	13.5 [8.75;20.0]	0.010
pH (med, [IQR])	7.35 [7.28;7.39]	7.35 [7.30;7.39]	7.32 [7.25;7.37]	0.007
Lactates (med, [IQR], mmoL/L)	2.10 [1.30;3.60]	2.00 [1.20;3.18]	2.50 [1.80;4.35]	<0.001
Base excess (med, [IQR], mmoL/L)	−3.43 [−6.74; −1.52]	−2.85 [−5.96;−1.12]	−5.38 [−8.93;−2.09]	0.002
Calcemia (med, [IQR], mmoL/L)	1.12 [1.07;1.16]	1.13 [1.08;1.16]	1.10 [1.06;1.15]	0.046

**Table 5:** Population characteristics with and without coagulopathy. SBP: systolic blood pressure; SpO_2_: oxygen saturation; GCS: Glasgow coma scale; MV: mechanical ventilation; ICU: intensive care unit.

	Patients Without TIC *n* = 227	Patient with TIC *n* = 160	Significance Level (*p*)
Age (med, [IQR], bpm)	39.0 [28.0;51.0]	39.0 [26.0;54.2]	0.965
Sex (*n*, %)			0.562
Female	43 (18.9%)	35 (21.9%)	
Male	184 (81.1%)	125 (78.1%)	
Prehospital parameters			
Heart rate (med, [IQR], bpm)	90.0 [75.5;110]	100 [86.5;120]	<0.001
SBP (med, [IQR], mmHg)	120 [103;131]	96.0 [80.0;120]	<0.001
SpO_2_ (med, [IQR], %)	99.0 [96.0;100]	98.0 [90.0;100]	0.007
Respiratory rate (med, [IQR], cycles/min)	20.0 [16.0;24.0]	16.5 [15.0;20.0]	0.126
GCS (med, [IQR])	15.0 [15.0;15.0]	15.0 [13.0;15.0]	<0.001
Crystalloid volume (med, [IQR, mL)]	500 [250;500]	500 [500;1000]	<0.001
Hemodynamic distress (*n*, %)			<0.001
Stable (group 3)	167 (73.9%)	46 (28.7%)	
Unstable (group 1)	12 (5.31%)	44 (27.5%)	
Stabilized (group 2)	47 (20.8%)	70 (43.8%)	
Scores			
MGAP (med, [IQR])	23.0 [22.0;25.0]	22.0 [11.0;23.0]	<0.001
RTS (med, [IQR])	7.84 [7.84;7.84]	7.84 [7.06;7.84]	<0.001
TRISS (med, [IQR])	3.80 [3.02;4.37]	3.10 [2.02;3.95]	<0.001
TASH (med, [IQR])	3.00 [1.00;6.00]	10.5 [6.00;15.0]	<0.001
IGS (med, [IQR])	16.0 [11.0;23.0]	29.0 [17.0;49.0]	<0.001
SI > 0.9 (med, [IQR])	71 (31.6%)	110 (69.2%)	<0.001
AIS thorax ≥ 3 (*n*, %)	106 (46.9%)	58 (36.2%)	0.048
AIS abdomen ≥ 3 (*n*, %)	26 (11.5%)	35 (21.9%)	0.009
AIS members/pelvis ≥3 (*n*, %)	23 (10.2%)	40 (25.0%)	<0.001
AIS spine ≥ 3 (*n*, %)	21 (12.0%)	6 (5.83%)	0.142
Pelvis			
Pelvic injury (*n*, %)	40 (17.6%)	73 (45.6%)	<0.001
Classification according to Tile (*n*, %)			<0.001
A	28 (12.3%)	49 (30.6%)	
B	5 (2.20%)	11 (6.88%)	
C	7 (3.08%)	13 (8.12%)	
Interventional management (*n*, %)			
Hemostatic surgery	53 (23.3%)	81 (50.6%)	<0.001
Surgery during the first 24 h	126 (55.5%)	95 (59.4%)	0.514
Embolization	17 (7.49%)	27 (16.9%)	0.007
Evolution			
ICU admission (*n*, %)	65 (28.6%)	116 (72.5%)	<0.001
ICU length of stay (med, [IQR], days)	0.00 [0.00;1.00]	3.00 [0.00;8.00]	<0.001
MV length of stay (med, [IQR], days)	0.00 [0.00;0.00]	1.00 [0.00;3.00]	<0.001
Death (*n*, %)	4 (1.76%)	20 (12.5%)	<0.001

**Table 6:** Logistic regression results.

Variable	Category	*n*	Odds Ratio (95% CI)	*p*-Value	VIF
Pelvic injury	No	262	Reference	–	1.027
	Yes	110	2.81 (1.63–4.89)	<0.001	
Hemoglobin (g/dL)	[10, 18]	317	Reference	–	1.104
	[4, 10]	55	4.27 (1.77–11.49)	0.002	
Base Excess (mmol/L)	Continuous	372	0.92 (0.87–0.97)	0.004	1.237
Shock Index	≤0.9	193	Reference	–	1.260
	> 0.9	179	1.94 (1.12–3.37)	0.017	
Volemic Expansion (mL)	[0, 500)	135	Reference	–	1.074
	[500, 1000)	142	1.28 (0.73–2.27)	0.391	
	[1000, 5500]	95	1.64 (0.85–3.16)	0.140	
Hemostatic surgery	No	241	Reference	–	1.154
	Yes	131	1.59 (0.91–2.75)	0.098	
ISS	≤25	227	Reference	–	1.283
	>25	145	1.70 (0.96–3.01)	0.069	
Number of lesions AIS ≥ 3	Continuous	372	0.70 (0.45–1.07)	0.098	1.368

**Model Performance:** Area Under the Curve (AUC): 0.812, Hosmer–Lemeshow test: *p* = 0.832. Overall model fit: Good calibration and discrimination. **Variables included:** Pelvic injury (qualitative), Hemoglobin (qualitative), Base excess (quantitative), Shock index (qualitative), Volemic expansion (qualitative), Damage control surgery (qualitative), ISS (qualitative), Number of lesions (quantitative). **Abbreviations:** CI, confidence interval; VIF, variance inflation factor; ISS, injury severity score; AIS, abbreviated injury scale.

## Data Availability

The data presented in this study are available on request from the corresponding author, subject to authorization by the relevant military authority.

## Supplementary Materials

The following supporting information can be downloaded at https://www.mdpi.com/article/10.3390/jcm15062365/s1: Figure S1: Details of missing values; Table S1: Main missing values; Table S2: Pelvic injury characteristics according to Tile classification.

## Abbreviations

The following abbreviations are used in this manuscript:

AIS	Abbreviated Injury Scale
aPTT	Activated Partial Thromboplastin Time
DCR	Damage Control Resuscitation
DCS	Damage Control Surgery
ED	Emergency Department
ICU	Intensive Care Unit
IGS/SAPS II	Simplified Acute Physiology Score II
INR	International Normalized Ratio
ISS	Injury Severity Score
MGAP	Mechanism–Glasgow Coma Scale–Age–Arterial Pressure Score
ODC	Orthopedic Damage Control
PT	Prothrombin Time
PTT	Partial Thromboplastin Time
RTS	Revised Trauma Score
SI	Shock Index
SpO_2_	Peripheral oxygen saturation
TASH	Trauma Associated Severe Hemorrhage score
TBI	Traumatic Brain Injury
TIC	Trauma-Induced Coagulopathy
TRISS	Trauma and Injury Severity Score
