# Supplementary material for: Association Between Pelvic Injury and Trauma-Induced Coagulopathy in Severe Trauma Patients: A Retrospective Single-Center Study
Source: J Clin Med. 2026 Mar 19;15(6):2365. doi: 10.3390/jcm15062365 (PMC13026385; doi:10.3390/jcm15062365)
Supplement: Supplementary file 1 [file jcm-15-02365-s001.zip › jcm-4057018-supplementary.pdf]

Figure S1 : Details of missing values

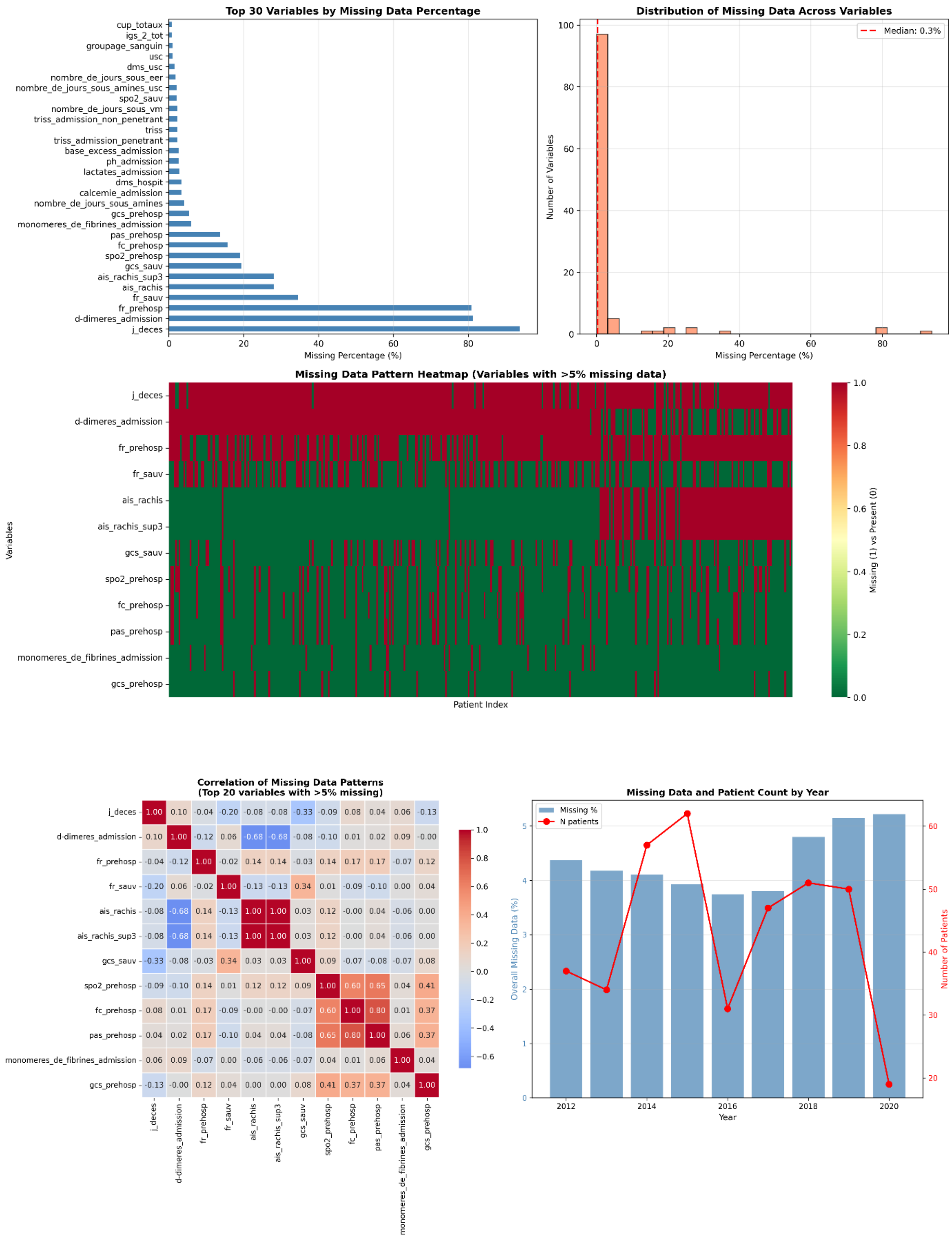

Table S1 Main missing values

Variable	Data_Type	Total_Observations	Missing_Count	Present_Count	Missing_Percentage
day_death	float64	388	364	24	93.81
d-dimeres_admission	float64	388	315	73	81.19
RR_prehosp	float64	388	314	74	80.93
RR_arrival	float64	388	134	254	34.54
ais_spine	float64	388	109	279	28.09
ais_spine_sup3	float64	388	109	279	28.09
gcs_arrival	float64	388	75	313	19.33
spo2_prehosp	float64	388	74	314	19.07
HR_prehosp	float64	388	61	327	15.72
SBP_prehosp	float64	388	53	335	13.66
Fibrine_monomeres_admission	float64	388	23	365	5.93
gcs_prehosp	float64	388	21	367	5.41
days_with_vasopressors	float64	388	16	372	4.12
calcemia_admission	float64	388	13	375	3.35
LOS_hospital	float64	388	13	375	3.35
lactate_admission	float64	388	11	377	2.84
ph_admission	float64	388	10	378	2.58
base_excess_admission	float64	388	10	378	2.58
triss_admission_penetrating	float64	388	9	379	2.32
triss	float64	388	9	379	2.32
triss_admission_blunt	float64	388	9	379	2.32
days_with_mechanical_ventilation	float64	388	9	379	2.32
spo2_admission	float64	388	8	380	2.06
days_with_vasopressors_MCU	float64	388	8	380	2.06
days_with_RRT	float64	388	7	381	1.8
LOS_MCU	float64	388	6	382	1.55
MCU	float64	388	4	384	1.03
blood_type	object	388	4	384	1.03
SAPS_2_tot	float64	388	3	385	0.77
platelet_concentrate_total	float64	388	3	385	0.77
dried_plamsa_total	float64	388	3	385	0.77
frozen_plasma_day_1	float64	388	3	385	0.77
LOS_ICU	float64	388	3	385	0.77

Table S2 : Pelvic injury characteristics according to Tile classification

Tile classification	A	B	C	p value
	N=77	N=17	N=20	
**Coagulopathy**				0.926
No	28 (36.4%)	5 (31.2%)	7 (35.0%)	
Yes	49 (63.6%)	11 (68.8%)	13 (65.0%)	
**ISS**	27.2 (10.6)	29.7 (10.0)	29.5 (8.66)	0.495
**Hb on admission**	12.0 (2.62)	10.7 (2.95)	10.6 (2.80)	0.068
**Base excess on admission**	-4.37 (6.54)	-6.58 (3.80)	-8.11 (5.56)	0.030
**Volemic expansion**	621 (577)	771 (695)	1155 (1179)	0.142
**Shock Index (SI)**				0.133
SI > 0.9	44 (57.1%)	12 (70.6%)	16 (80.0%)	
SI ≤ 0.9	33 (42.9%)	5 (29.4%)	4 (20.0%)	
**Hemostatic surgery**				0.049
No	50 (64.9%)	9 (52.9%)	7 (35.0%)	
Yes	27 (35.1%)	8 (47.1%)	13 (65.0%)	
**Number of lesions AIS≥3**	77 (100%)	17 (100%)	20 (100%)	.
**Facial AIS≥3**				0.466
No	53 (68.8%)	14 (82.4%)	13 (65.0%)	
Yes	24 (31.2%)	3 (17.6%)	7 (35.0%)	
**Thorax AIS≥3**				0.586
No	70 (90.9%)	15 (88.2%)	17 (85.0%)	
Yes	7 (9.09%)	2 (11.8%)	3 (15.0%)	
**Abdomen AIS≥3**				0.629
No	49 (92.5%)	9 (90.0%)	13 (100%)	
Yes	4 (7.55%)	1 (10.0%)	0 (0.00%)	
**Spine AIS≥3**				0.049
No	50 (64.9%)	9 (52.9%)	7 (35.0%)	
Yes	27 (35.1%)	8 (47.1%)	13 (65.0%)	
